# Characterisation of Typical Enteropathogenic *Escherichia coli* (tEPEC) and *bfpA* Genes From Five Bat Species Reveals Australian *Pteropus* Spp. Are Natural tEPEC Hosts

**DOI:** 10.1002/mbo3.70403

**Published:** 2026-09-07

**Authors:** Fiona K. McDougall, Laura A. Pulscher, Wayne S. J. Boardman, Karrie Rose, Mariel Fulham, Michelle L. Power

**Affiliations:** ^1^ School of Natural Sciences, Faculty of Science and Engineering Macquarie University Sydney New South Wales Australia; ^2^ School of Veterinary Science, Faculty of Science University of Sydney Sydney New South Wales Australia; ^3^ Department of Microbiology, Immunology, and Pathology Colorado State University Fort Collins Colorado USA; ^4^ School of Animal and Veterinary Sciences, College of Sciences Adelaide University Adelaide South Australia Australia; ^5^ Australian Registry of Wildlife Health Taronga Conservation Society Australia Sydney New South Wales Australia

**Keywords:** bfpA allele, diarrhoeal *Escherichia coli*, flying fox, fruit bat, one health

## Abstract

Humans have historically been considered the only natural host of typical enteropathogenic *Escherichia coli* (tEPEC), a cause of human infantile diarrhoea. Recent findings of bat‐specific tEPEC in two Australian bat species (*Pteropus poliocephalus* and *Pteropus conspicillatus*) revealed that these *Pteropus* spp. are also tEPEC hosts. tEPEC pathogenicity is associated with key virulence factors including intimin (*eae*) and the bundle‐forming pilus (*bfp*) operon, which contains the *bfpA* gene. This study characterised 63 tEPEC isolates from five Australian *Pteropus* spp.; four from mainland Australia (*P. poliocephalus*, *P. conspicillatus, P. alecto* and *P. scapulatus*) and one from Christmas Island (*P. natalis)*. The 63 tEPEC isolates included 10 novel tEPEC strains and eight previously identified *Pteropus* tEPEC strains. Faecal DNA samples (*n* = 386) from the five Australian *Pteropus* spp. were screened for *eae* and *bfpA* genes to identify tEPEC‐positive samples. The estimated true prevalence of tEPEC ranged from 14.2% to 37.0% across the five *Pteropus* spp. Typing of 119 *bfpA* alleles (63 tEPEC isolates and 56 faecal DNA samples) from the five *Pteropus* spp. identified 27 *bfpA* allele types, 24 of which belonged to bat‐specific *bfpA* lineages. Bat‐specific *bfpA* alleles were shared between tEPEC strains, *Pteropus* spp. and across regions, including the geographically isolated *P. natalis* endemic to Christmas Island. This study reveals that diverse bat‐specific tEPEC strains and *bfpA* types have evolved in *Pteropus* spp. and have been circulating among populations for an extensive period, thereby confirming that all Australian *Pteropus* spp. are natural tEPEC hosts.

## Graphical abstract

This study revealed that Australia's five *Pteropus* bat species are all natural hosts of typical Enteropathogenic *Escherichia coli* (tEPEC)—historically considered a human‐specific pathogen. Across the five *Pteropus* spp., tEPEC prevalence ranged between 14.2% and 37.0%, with 18 bat‐specific tEPEC strain types and 24 bat‐specific *bfpA* allele types identified. Diverse bat‐specific tEPEC strains and *bfpA* types have evolved in Australian *Pteropus* spp., including the now geographically isolated Christmas Island flying fox, and have been circulating among populations for an extensive period.

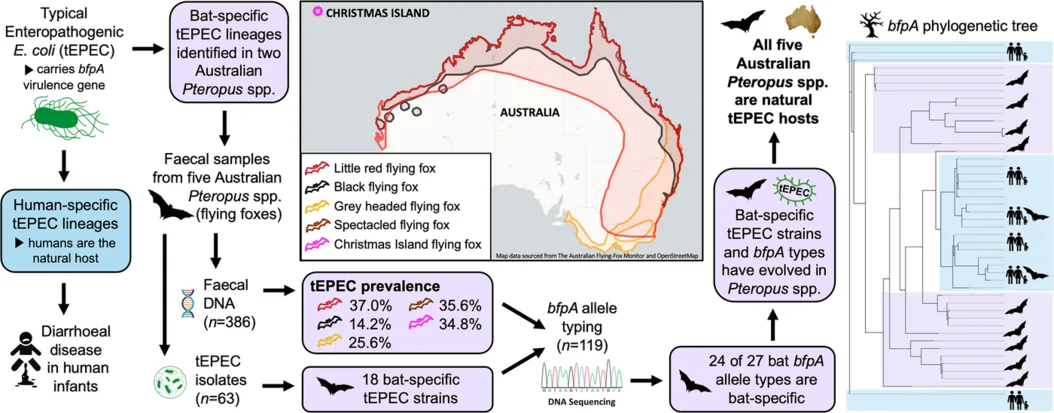

## 1 Introduction

Bats are considered the evolutionary origin of many infectious agents of humans, including multiple highly infectious viruses (Liu et al. 2024; Wu et al. 2016), the haemoprotozoan parasites *Trypanosoma* (Clément et al. 2020) and haemosporida (malarial parasites) (Schaer et al. 2013), and the bacterial pathogen *Bartonella* (McKee et al. 2021). Whereas humans are considered to be the evolutionary origin of multiple highly adapted pathogens that specifically infect humans, including *Mycobacterium tuberculosis* (Gagneux 2018), variola virus (smallpox) (Duggan et al. 2016) and typical enteropathogenic *Escherichia coli* (tEPEC) (Lacher et al. 2007). Humans continue to be recognised as the primary host and reservoir for tEPEC (Nataro and Kaper 1998), with tEPEC rarely detected in non‐human hosts (Chandran and Mazumder 2013; Sanches et al. 2017). However, mounting evidence has demonstrated that bats from the Pteropodidae family are also natural tEPEC hosts of bat‐specific tEPEC lineages (F. McDougall et al. 2023; F. K. McDougall and Power 2025; Nowak et al. 2017).

In 2017, three tEPEC strains with novel sequence types (ST) were described in two bat species (*Myonycteris torquata* and *Epomops franqueti*) in the Republic of Congo (Nowak et al. 2017). Subsequently, diverse tEPEC strains were identified in two Australian bat species, *Pteropus poliocephalus* (grey‐headed flying fox; GHFF) and *Pteropus conspicillatus* (spectacled flying fox; SFF) (F. McDougall et al. 2023; F. K. McDougall and Power 2025). The Australian bat tEPEC were also predominantly novel strain types, which belonged to bat‐specific tEPEC lineages representing five distinct clades (F. McDougall et al. 2023; F. K. McDougall and Power 2025). Consistent detection and a high prevalence of tEPEC were observed in *P. poliocephalus* over extensive geographical ranges and time periods, indicating that *P. poliocephalus* is a natural tEPEC host (F. McDougall et al. 2023).

In humans, tEPEC are associated with prolonged diarrhoea and mortality of infants, particularly in low‐ and middle‐income countries (Kaper et al. 2004; Levine et al. 2020). The intestinal pathogenicity of tEPEC is determined by two key virulence factors: a locus for enterocyte effacement (LEE) pathogenicity island and the EPEC adherence factor plasmid (pEAF) (Trabulsi et al. 2002). LEE encodes 41 genes, including *eae*, which encodes intimin (Ingle et al. 2016). Multiple variants of LEE have evolved, with *eae* allele types largely conserved within distinct EPEC lineages (Ingle et al. 2016; Lacher et al. 2007; Müller et al. 2009). The pEAF plasmid encodes the bundle‐forming pilus (*bfp*) operon and plasmid‐encoded regulators (*per*) (Levine et al. 1985). Multiple variants of pEAF and *bfpA* alleles have also evolved in human‐sourced tEPEC; however, pEAF and *bfpA* are not highly conserved within tEPEC lineages (Lacher et al. 2007). The human‐associated *bfpA* alleles belong to two distinct lineages, designated as the alpha and beta *bfpA* clades (Lacher et al. 2007). Ten novel *bfpA* alleles were identified in bat‐sourced tEPEC, all of which were most closely related to the human beta *bfpA* clade (F. McDougall et al. 2023; F. K. McDougall and Power 2025). Further phylogenetic analysis of the entire *bfp* operon, which encodes 14 genes, determined that bat‐sourced *bfp* operons belong to a lineage that is distinct from human‐sourced *bfp* operons (F. K. McDougall and Power 2025). Additionally, phylogenetic analysis and *eae* allele typing of tEPEC strains from Australian bats determined that bat tEPEC shared phylogenetic lineages with several human‐associated aEPEC lineages, rather than with human‐associated tEPEC lineages, and acquired bat‐specific *bfp* operons independently (F. McDougall et al. 2023). This shared evolutionary origin, the diversification of *bfpA* alleles in bats and humans and the apparent absence of pathogenicity in bats, suggests that bats may be the evolutionary origin of tEPEC (F. McDougall et al. 2023; F. K. McDougall and Power 2025).

To further evaluate the tEPEC host status of Australian *Pteropus* spp., we investigated the occurrence of tEPEC in three additional *Pteropus* spp., *Pteropus alecto* (black flying fox; BFF), *Pteropus scapulatus* (little red flying fox; LRFF) and *Pteropus natalis* (Christmas Island flying fox; CIFF) and expanded the current knowledge of tEPEC in *P. poliocephalus* (GHFF) and *P. conspicillatus* (SFF). This study examined the types of tEPEC strains in these *Pteropus* spp., the diversity of *bfpA* alleles, and the evolutionary relationships between human and bat *bfpA* types. The study aimed to determine if all five Australian *Pteropus* spp. are natural hosts for bat‐specific tEPEC lineages.

## 2 Materials and Methods

### 2.1 Pteropus Spp. in This Study

**Figure 1:**
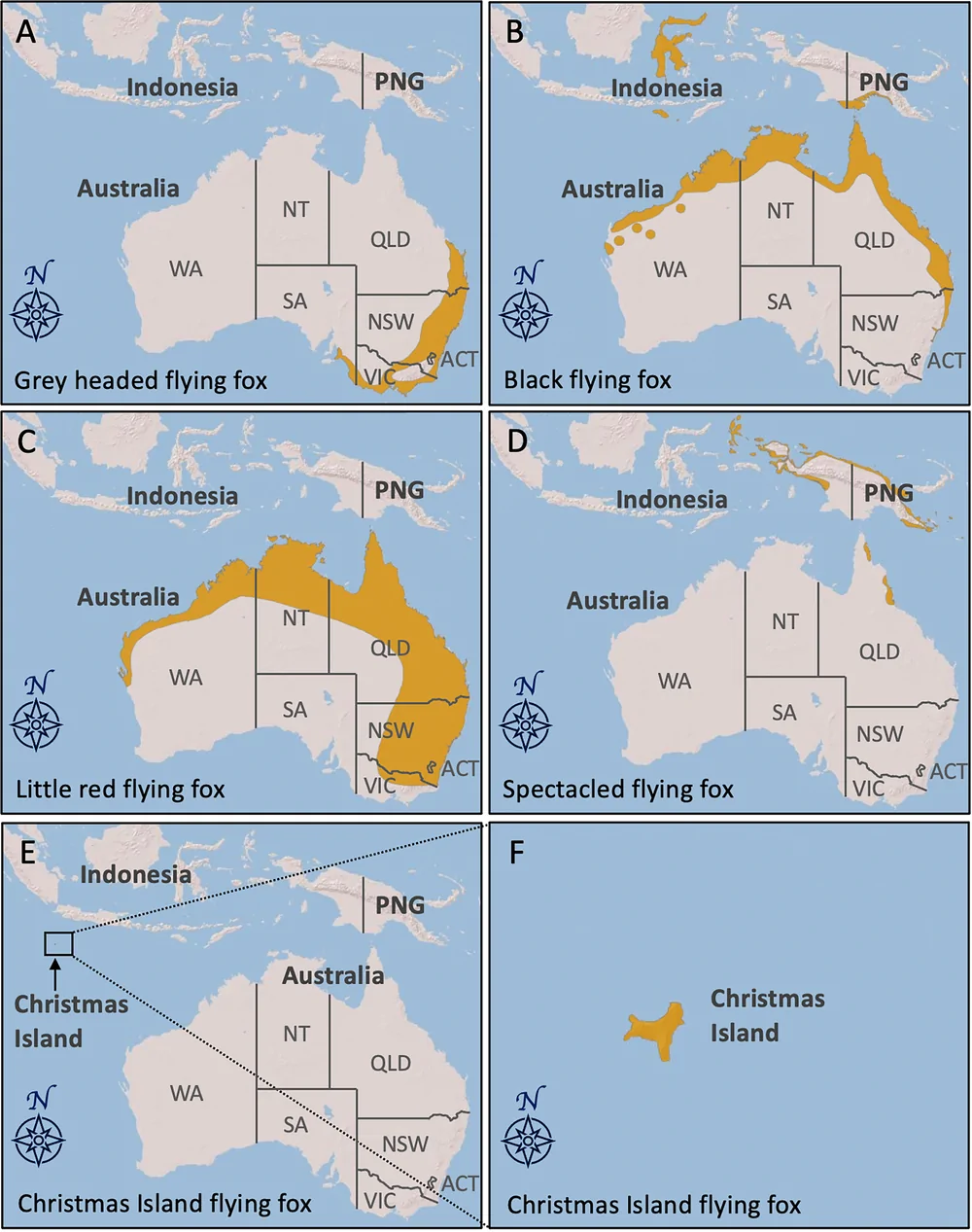
Maps showing the distribution of five Australian *Pteropus* spp.; (A) Grey‐headed flying fox, GHFF (*P. poliocephalus*), (B) Black flying fox, BFF (*P. alecto*), (C) Little red flying fox, LRFF (*P. scapulatus*), (D) Spectacled flying fox, SFF (*P. conspicillatus*), (E, F) Christmas Island flying fox, CIFF (*P. natalis*). Map images sourced from the International Union for Conservation of Nature (IUCN) Red List of Threatened Species (IUCN 2023) and modified under an IUCN User Agreement and license for scientific analyses and research usage. Mainland Australian States and Territories: ACT, Australian Capital Territory; NSW, New South Wales; NT, Northern Territory; QLD, Queensland; SA, South Australia; VIC, Victoria; WA, Western Australia. PNG, Papua New Guinea.

Five *Pteropus* spp. were included in this study: *P. poliocephalus* (GHFF), *P. alecto* (BFF), *P. scapulatus* (LRFF), *P. conspicillatus* (SFF) and *P. natalis* (CIFF). Four of the five species (GHFF, BFF, LRFF and SFF) are found on the Australian mainland (Figure 1a,d) with variable endemicity and ranges (Figure 1a,d). The CIFF is endemic to Christmas Island, an Australian external territory located in the Indian Ocean approximately 380 km south of Java, Indonesia, and 1500 km north‐west of the Australian mainland (Figure 1e,f).

### 2.2 Faecal Sample Collection

Faecal samples were collected from five *Pteropus* spp. (BFF, CIFF, GHFF, LRFF and SFF) for *E. coli* culture (Table S1) and/or DNA extraction (Table S2). The faecal samples were collected specifically for this study or provided to this study from other researchers (Tables S1 and S2) (F. McDougall et al. 2023; F. K. McDougall et al. 2024; Schiller et al. 2016). Sampled bats were either free‐living wild bats (hereon referred to as ‘wild’ bats) or injured/orphaned wild bats taken into care by wildlife hospitals or wildlife rescue and rehabilitation organisations (hereon referred to as bats ‘in‐care’). Sampling sites were located in three mainland Australian states; New South Wales (NSW), Queensland (QLD) and South Australia (SA), and in the Australian external territory of Christmas Island (Tables S1 and S2). Faecal samples were collected from clean plastic drop sheets or materials placed under bats in cages or roosts using a sterile swab which was transferred into Cary‐Blair media (FecalSwab, COPAN, Brescia, Italy), or faeces were collected directly into a sterile specimen container. Following collection, all faecal samples were stored at 4°C until processed for *E. coli* culture and/or DNA extraction, except CIFF samples, which were frozen at −80°C prior to transfer from Christmas Island to mainland Australia.

### 2.3 Isolation and Identification of Pteropus tEPEC

A total of 63 bat tEPEC isolates from five *Pteropus* hosts (BFF, CIFF, GHFF, SFF and LRFF) were available for analyses in this study (Table S1). The 63 bat tEPEC comprised 30 isolates obtained from 370 faecal samples collected specifically for this study (wild/in‐care BFF, CIFF, LRFF and SFF, and GHFF in‐care) and 33 isolates collected from wild GHFF as part of a previous study (Table S1) (F. McDougall et al. 2023).

The *E. coli* were isolated from *Pteropus* faecal samples using Chromocult Coliform Agar (Merck Millipore, Massachusetts, USA) and cell lysates were used as the DNA template for PCRs performed in this study, as previously described (F. McDougall et al. 2023). PCRs comprised a duplex PCR targeting the *eae* and *bfpA* genes to identify tEPEC isolates (F. McDougall et al. 2023) and a phylotyping PCR using the Clermont method to assign *E. coli* isolates to a phylogroup (Clermont et al. 2013) as previously described (F. McDougall et al. 2022). A full‐length *bpfA* PCR was developed to allow for deeper characterisation of tEPEC isolates (see below).

### 2.4 bfpA Allele Typing of Pteropus tEPEC

To amplify the entire *bfpA* allele, primers were designed using the Primer BLAST tool (https://www.ncbi.nlm.nih.gov/tools/primer-blast/) and nine full‐length *bfp* operon sequences (GenBank Accession Numbers OM925979 to OM925987) representative of GHFF tEPEC isolates (F. McDougall et al. 2023). The primer sequences, FFbfpAF 5′ GCGTGTCTTTTTTAGTTTTAAGATTATTC 3′ and FFbfpAR 5′ GCATCATCCAGCGACATATCC 3′ produce an amplicon of approximately 1008 bp. PCRs were performed using GoTaq Green Master Mix (Promega, Madison, USA), primers at 0.5 μM each and 3 μL *E. coli* DNA (cell lysate). PCR cycling conditions were 94°C 3 min; 40 cycles of 94°C 30 s, 58°C 30 s, 72°C 1 min 30 s; 72°C 5 min.

The *bfpA* amplicons were purified using the MinElute PCR Purification Kit (Qiagen, Hilden, Germany) and underwent Sanger sequencing at The Ramaciotti Centre for Genomics (Sydney, NSW, Australia) using BigDye Terminator chemistry version 3.1 and an ABI 3730 Capillary Sequencer (Applied Biosystems, Foster City, USA). The sequences were checked for quality using Geneious Prime software (Biomatters Limited, Auckland, New Zealand), and assigned to a *bfpA* allele type using a reference library containing 28 *bfpA* reference alleles, 18 of which were obtained from GenBank (Table S3) and 10 were *bfpA* allele types previously identified in bat‐sourced tEPEC isolates (GenBank Accession Numbers OM925979 to OM925987, PV285312 and PV285313) (Table S3) (F. McDougall et al. 2023; F. K. McDougall and Power 2025). The bat *bfpA* sequences were also submitted for BLASTn searches (https://blast.ncbi.nlm.nih.gov/Blast.cgi) to identify the closest sequence matches to both reference and undefined *bfpA* allele types. Representative sequences for all *Pteropus bfpA* allele types have been uploaded to GenBank under accession numbers PV285308 to PV285309, PV2853011, PV285314 to PV285327 and PV285329 to PV285349 (individual accession numbers are listed in Table S4).

### 2.5 Genome Fingerprinting PCRs of tEPEC Isolates From Pteropus Spp

To determine if tEPEC isolates carrying the same *bfpA* allele type were the same *E. coli* strain type, genome fingerprints were obtained using enterobacterial repetitive intergenic consensus (ERIC) PCR (Versalovic et al. 1991) and CGG‐PCR (targets the (CGG)_4_ repeat region) (Adamus‐Bialek et al. 2009). Fifteen previously characterised *Pteropus* tEPEC isolates representing 11 strains (BioProject PRJNA613581) were included as reference strains in genome fingerprinting PCRs (Table S5) (F. McDougall et al. 2023; F. K. McDougall and Power 2025).

Genomic DNA was extracted from tEPEC isolates using the ISOLATE II Genomic DNA Kit (Bioline, London, UK) and used as the template for PCRs. Genomic DNA concentrations were determined using a Qubit dsDNA BR assay kit (Invitrogen, Waltham, MA, USA). Both genome fingerprinting PCRs were performed using GoTaq Green Master Mix (Promega, Madison, WI, USA) with PCR conditions as previously described (F. McDougall et al. 2022). Isolate DNA fingerprints were visualised using 2% agarose gel electrophoresis with SYBR Safe DNA Stain (Invitrogen, Waltham, MA, USA).

### 2.6 tEPEC Prevalence in Pteropus Faecal DNA Samples

Faecal DNA extracts (*n* = 386) derived from *Pteropus* faeces (BFF *n* = 59, CIFF *n* = 24, GHFF *n* = 208, LRFF *n* = 48 and SFF *n* = 47) were used to estimate tEPEC prevalence. Of the 386 faecal DNA samples, 210 were sourced from a BioBank (F. K. McDougall et al. 2024) and the remaining 175 were collected for this study and extracted using the Bioline ISOLATE Faecal DNA Kit (Bioline, London, UK) (Table S2). Samples were obtained from bats in‐care (sick or injured adults and pups) and wild bats (adults and pups) (Table S2). For simplification, the term ‘adults’ includes adults and sub‐adults, and the term ‘pups’ includes pre‐weaned pups and weaned pups/juveniles.

The presence of tEPEC in faecal DNA samples was determined using a tEPEC‐specific multiplex PCR which targeted the *eae* and *bfpA* genes, and incorporated the *chuA* gene as an internal control (F. McDougall et al. 2023) to confirm the presence *E. coli* from phylogroups B2, D, E, F or G (Clermont et al. 2013). Faecal DNA samples which failed to amplify for *eae, bfpA* and *chuA* in the tEPEC‐specific multiplex PCR underwent a 16S rRNA PCR to confirm PCR competency using universal eubacterial primers f27 and r1492 (Lane 1991), as previously described (F. McDougall et al. 2023). The prevalence estimates and 95% confidence intervals were calculated for each of the five *Pteropus* populations (GHFF in‐care, BFF, LRFF, SFF and CIFF) using the Wilson score method with the epiR package (2.0.85) (Stevenson and Sergeant 2025) in R (4.5.1) (https://CRAN.R-project.org/package=epiR) and RStudio (2025.05.1 + 513). PCR sensitivity was estimated as 95.7% (calculated using tEPEC culture results and corresponding faecal DNA tEPEC PCR results) and specificity was calculated as 100% (based on confirmation of all faecal DNA tEPEC‐positives by DNA sequencing of the *bfpA* allele) (Supporting Information S1: data 1).

### 2.7 bfpA Allele Typing of tEPEC Positive Faecal DNA Samples

The full‐length *bfpA* PCR was used to characterise *bfpA* amplicons detected in faecal DNA samples, as described above for tEPEC isolates, with the following modifications: AllTaq Master Mix (Qiagen, Hilden, Germany) replaced GoTaq Green Master Mix (Promega, Madison, USA), and 3 uL faecal DNA extracts were used. Representative sequences for all bat *bfpA* allele types identified in faecal DNA have been uploaded to GenBank (Table S4).

### 2.8 Phylogenetic Inference of bfpA Alleles From Pteropus Spp

Phylogenetic analysis of bat *bfpA* alleles (*n* = 27, Table S4) and *bfpA* reference alleles sourced from GenBank (*n* = 20, Table S3) was performed using RAxML tree builder in Geneious Prime software with nucleotide model GTR CAT and rapid hill‐climbing algorithm (Biomatters Limited, Auckland, New Zealand). The 20 reference *bfpA* alleles were sourced from human and non‐bat animal tEPEC (*n* = 18) and bat‐sourced tEPEC which were detected in a previous study (*n* = 2) (Table S3).

Alignments of nucleotide and translated amino acid sequences of all *bfpA* allele types from humans, *Pteropus* spp. and other animals were performed using Geneious Prime software (Biomatters Limited, Auckland, New Zealand).

## 3 Results

### 3.1 bfpA Allele Typing in tEPEC Isolates

A total of 63 tEPEC isolates from all five *Pteropus* spp. (BFF *n* = 1, CIFF *n* = 6, GHFF *n* = 49, LRFF *n* = 2 and SFF *n* = 5) were characterised (Table S1). Fourteen different *bfpA* allele types were detected across the 63 *Pteropus* tEPEC isolates (Table S4). Seven of the fourteen *bfpA* alleles matched those previously found in bats (Tables S3 and S4). Among the remaining seven alleles, three differed by 1–3 single nucleotide polymorphisms (SNPs) to previously identified *bfpA* alleles and four represented novel *bfpA* types (designated FF‐7.1, FF‐8.1, FF‐9.1 and FF‐10.1) (Table S4).

### 3.2 tEPEC Strain Types and bfpA Allele Associations

**Figure 2:**
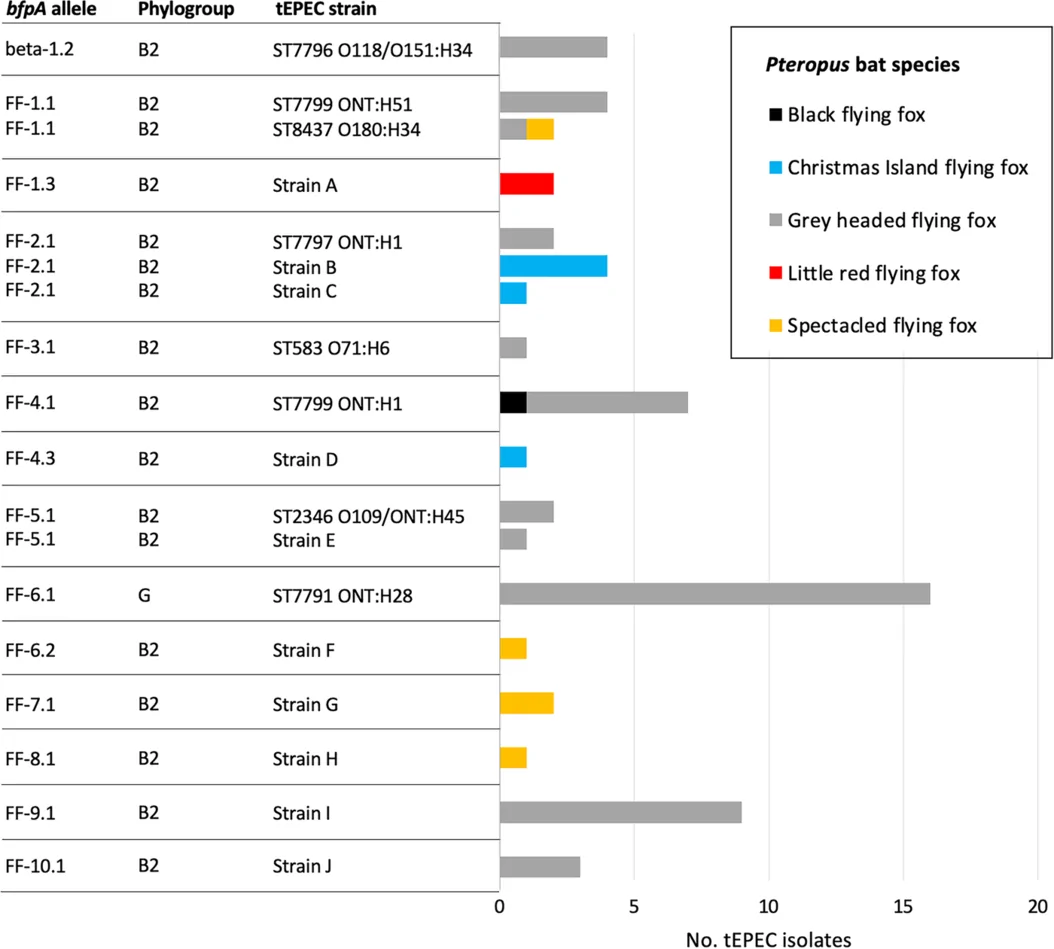
Molecular characterisation of 63 tEPEC isolates from five *Pteropus* spp. (representing 18 tEPEC strain types including 10 novel strains). All individual tEPEC isolate data is available in Supporting data 1. *Pteropus* spp.: Black flying fox, BFF (*P. alecto*); Christmas Island flying fox, CIFF (*P. natalis*); Grey‐headed flying fox, GHFF (*P. poliocephalus*); Little red flying fox, LRFF (*P. scapulatus*); Spectacled flying fox, SFF (*P. conspicillatus*).

The combined analysis of phylotyping, *bfpA* allele typing and genomic fingerprinting (ERIC PCR and CGG‐PCR) revealed that the 63 tEPEC isolates belonged to 18 distinct tEPEC strain types (Figure 2 and Supporting Information S1: data 1). Of the 18 strain types, eight strains represented by 38 isolates could be assigned to an ST and serotype based on comparison to reference isolates (Table S5). Ten novel strains represented by 25 isolates were identified and arbitrarily designated tEPEC Strains A–J (Figure 2). The most frequently detected tEPEC strains were the known ST7791 ONT:H28 (*n* = 16) and ST7799 ONT:H1 (*n* = 7), and the novel Strain I (*n* = 9) (Figure 2). Of the 63 tEPEC isolates, 16 were designated phylogroup G (all ST7791 ONT:H28), and the remaining 47 tEPEC were designated phylogroup B2 (Figure 2).

The majority (16 of 18) tEPEC strains were detected in only one *Pteropus* species, with two tEPEC strains being detected in two different *Pteropus* spp.; ST8437 in GHFF and SFF, and ST7799 in BFF and GHFF (Figure 2). Seven of 18 tEPEC strains were detected in bats at multiple locations, with periods between detections ranging from 1 month to 26 months (Supporting data 1). Notably, these multiple location detections included the phylogroup G strain ST7791 ONT:H28 (SA February 2018 and NSW April 2018), that was previously detected in NSW in 2015 (Enterobase Barcode ESC_HA7318AA, Table S5).

Eleven of 18 tEPEC strains carried a distinct *bfpA* allele type that was not shared with any other tEPEC strain types (Figure 2). Five of 18 tEPEC strains shared a *bfpA* allele type with at least one other tEPEC strain type (Figure 2). Notably, the FF‐2.1 *bfpA* allele was detected in three tEPEC strains: one tEPEC strain isolated from GHFF on mainland Australia (ST7797 ONT:H1) and two different tEPEC strains from CIFF located on Christmas Island (Strain B and Strain C) (Figure 2).

### 3.3 Faecal DNA Prevalence of tEPEC

Screening for tEPEC in *Pteropus* faecal DNA using a tEPEC‐specific multiplex PCR revealed that 25.9% (100 of 386) were positive for the three target loci; *eae, bfpA* and *chuA* (Table 1). The estimated true prevalence of tEPEC across all five *Pteropus* spp. ranged from 14.2% (BFF) to 37.0% (LRFF) (Table 1). Notably, the estimated true prevalence of tEPEC in GHFF adults in‐care and GHFF pups in‐care were similar, 25.8% and 25.5% respectively (Table 1).

**Table 1:** Apparent prevalence of tEPEC positive faecal DNA samples (as determined by the presence of the eae and bfpA virulence genes) and estimated true prevalence of tEPEC with 95% confidence intervals (calculated using the epiR package in R with sensitivity = 95.7% and specificity = 100%) in five Pteropus spp. of bats. Pteropus spp.: Black flying fox, BFF (P. alecto); Christmas Island flying fox, CIFF (P. natalis); Grey headed flying fox, GHFF (P. poliocephalus); Little red flying fox, LRFF (P. scapulatus); Spectacled flying fox, SFF (P. conspicillatus). Adults; includes adults and sub‐adults. Pups; includes pre‐weaned pups and weaned pups/juveniles. CI; Confidence interval.

	GHFF (Adults)	GHFF (Pups)	BFF (Adults + Pups)	LRFF (Adults + Pups)	SFF (Adults + Pups)	CIFF (Adults + Pups)	TOTAL (All five *Pteropus* spp.)
No. faecal Samples	89	119	59	48	47	24	386
No. tEPEC positive	22	29	8	17	16	8	100
Apparent prevalence	24.7%	24.4%	13.6%	35.4%	34.0%	33.3%	25.9%
Estimated true prevalence	25.8%	25.5%	14.2%	37.0%	35.6%	34.8%	
95% CI	17.7% – 36.2%	18.3% – 34.3%	7.4% – 25.6%	24.5% – 51.8%	23.2% – 50.5%	18.8% – 55.7%	

### 3.4 bfpA Allele Types in Faecal DNA From Pteropus Spp

For tEPEC‐positive faecal DNA samples from which no corresponding tEPEC isolates were cultured (*n* = 76), sequencing of the full‐length *bfpA* PCR amplicons determined the *bfpA* allele type in 56 faecal DNA samples across all five *Pteropus* spp. (BFF *n* = 7, CIFF *n* = 3, GHFF *n* = 28, LRFF *n* = 11 and SFF *n* = 7) (Supporting Information S1: data 1).

Twenty‐one different *bfpA* allele types were detected across the 56 *Pteropus* faecal DNA samples, which included eight *bfpA* alleles previously identified in *Pteropus* tEPEC isolates (Table S4). Nine of 21 faecal *bfpA* alleles differed by 1 − 8 SNPs to previously identified alleles in tEPEC isolates from *Pteropus* spp. (*n* = 8) and humans (*n* = 1) (Table S4). Four of 21 faecal *bfpA* alleles were novel types (designated FF‐11.1, FF‐12.1, FF‐13.1 and FF‐14.1), which showed 90.8%–95.7% identity to reference or undefined *bfpA* alleles in GenBank (Table S4).

The remaining 20 full‐length *bfpA* PCR amplicons returned mixed nucleotide traces, indicating carriage of multiple *bfpA* alleles, thus identification of specific *bfpA* allele types for these tEPEC‐positive faecal DNA samples was not possible (Supporting Information S1: data 1).

### 3.5 Phylogeny of bfpA Alleles From Pteropus Spp., Humans and Other Animals

**Figure 3:**
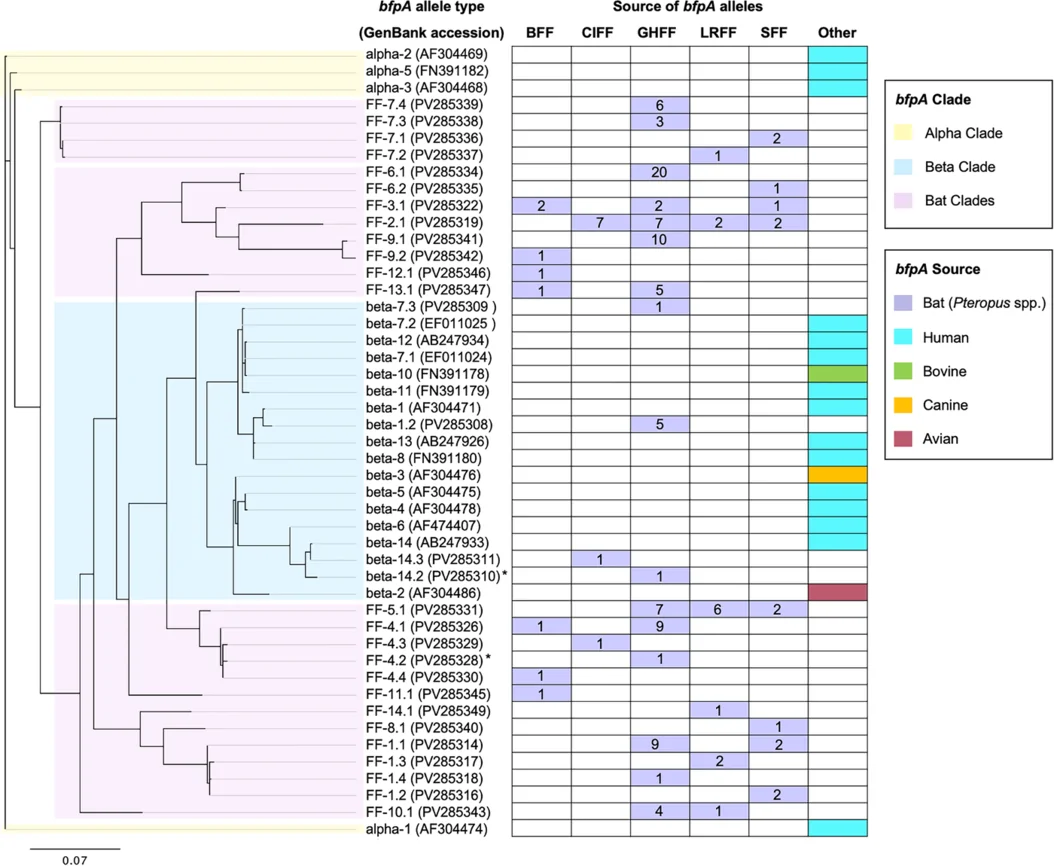
Left: Phylogenetic tree constructed using RAxML with 27 *Pteropus bfpA* allele types identified in this study (Table S4) and 20 reference *bfpA* alleles sourced from tEPEC in humans (*n* = 15), non‐bat animals (*n* = 3) and from previous bat studies (*n* = 2) (Table S3). Asterix (*) indicates the two reference *bfpA* alleles (beta‐14.2 and FF‐4.2) only identified in tEPEC from *Pteropus* spp. in a previous study (F. McDougall et al. 2023) (Table S3). Right: Host sources for the 119 full‐length *Pteropus bfpA* alleles (this study),15 *bfpA* alleles (previous studies) (Tables S3 and S5), and 18 reference *bfpA* alleles from human tEPEC (*n* = 15) and non‐bat animals (*n* = 3) (Table S3). The numbers inside rectangles indicate the number of times a *bfpA* allele has been detected in each *Pteropus* species. *Pteropus* spp.: Black flying fox, BFF (*P. alecto*); Christmas Island flying fox, CIFF (*P. natalis*); Grey‐headed flying fox, GHFF (*P. poliocephalus*); Little red flying fox, LRFF (*P. scapulatus*); Spectacled flying fox, SFF (*P. conspicillatus*).

Across the five *Pteropus* spp., 119 full‐length *bfpA* allele sequences were obtained (63 tEPEC isolates and 56 tEPEC*‐*positive faecal DNA samples) which comprised 27 *bfpA* allele types (Table S4). Phylogenetic analysis of the 27 bat *bfpA* allele types and 20 reference *bfpA* alleles (Table S3), placed 24 of 27 bat *bfpA* alleles in bat‐specific lineages (designated clades FF‐1 to FF‐14) and three bat *bfpA* alleles clustered with human‐associated beta clade *bfpA* alleles (Figure 3 and Table S4). All bat‐specific *bfpA* allele lineages showed ≤ 93.71% identity to reference and undefined *bfpA* allele types in GenBank, except bat *bfpA* clade FF‐7 which showed 96.91%–97.08% identity to alpha clade *bfpA* alleles (Figure 3 and Table S4).

Host source data for the 119 full‐length *bfpA* alleles revealed that seven *bfpA* alleles were shared by multiple *Pteropus* spp. (Figure 3). Notably, the FF‐2.1 *bfpA* allele was detected in four *Pteropus* spp., including three mainland Australian species (GHFF, LRFF and SFF) and CIFF (endemic to Christmas Island) (Figure 3). The remaining 20 bat *bfpA* allele types were detected in only one *Pteropus* sp. (Figure 3). BLASTn searches determined that none of the 27 *Pteropus bfpA* allele types has been reported in tEPEC from non‐bat hosts.

Nucleotide alignment of the 27 *Pteropus bfpA* alleles (Table S4) and 20 reference *bfpA* alleles (Table S3) identified conserved regions that largely occurred in the first‐third (N‐terminal) and variable regions predominantly in the last two‐thirds (C‐terminal) of bat *bfpA* allele sequences (Figure S1). The conserved and variable regions in the bat *bfpA* allele sequences correlated with conserved and variable regions observed in human‐ and non‐bat‐associated *bfpA* alleles (Figure S1).

### 3.6 Temporal and Geographical Distribution of bfpA Alleles in Five Pteropus Spp

**Figure 4:**
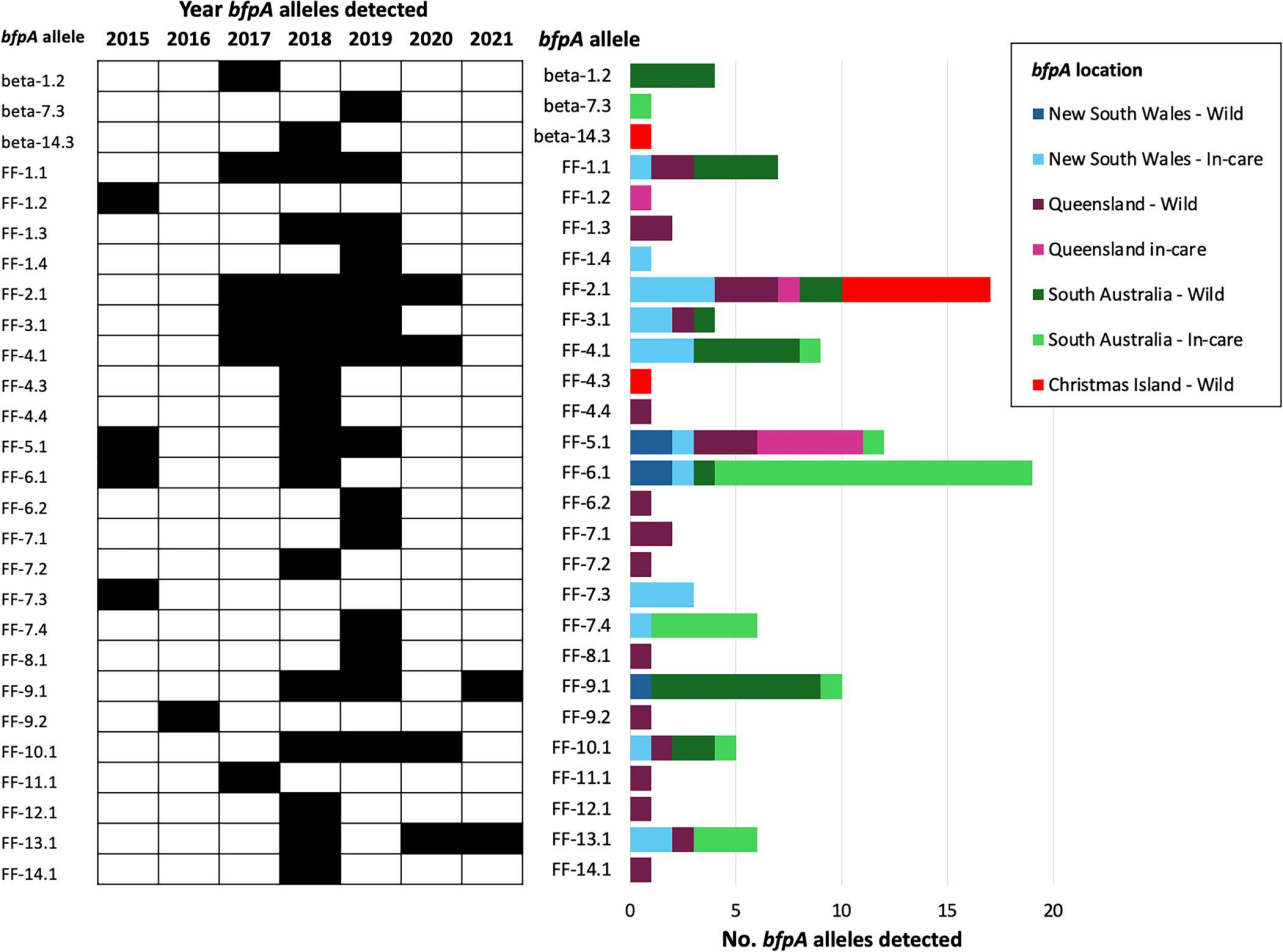
Temporal and geographical distribution of 119 full length *bfpA* alleles from five *Pteropus* spp. over a six‐year period (April 2015 and March 2021). Black cells indicate *bfpA* allele detection in the corresponding year.

Analysis of the temporal, geographical and host source data for the 119 full‐length *bfpA* alleles determined that bat‐specific *bfpA* allele types were detected in both wild and in‐care *Pteropus* across NSW, QLD, SA and Christmas Island, over a 6‐year period (April 2015 to March 2021) (Figure 4 and Supporting Information S1: data 1). Seven *bfpA* allele types were identified in both wild and in‐care *Pteropus* from the same location (Figure 4). Over the 6‐year period, 10 *bfpA* alleles were detected in multiple years and 17 alleles in a single year (Figure 4). Notably, the *bfpA* allele FF‐2.1 was detected in all four states and territories (NSW, QLD, SA and Christmas Island) over a 3‐year period (February 2017 to January 2020) (Figure 4).

## 4 Discussion

This study demonstrated that tEPEC isolates, historically considered to be human‐specific, occur in five *Pteropus* spp. from Australia, providing further evidence that bats are natural tEPEC hosts. The *Pteropus* tEPEC isolates comprised diverse strain types and novel *bfpA* alleles, which is consistent with the characteristics of tEPEC previously reported in GHFF and SFF from Australia (F. McDougall et al. 2023; F. K. McDougall and Power 2025). Collectively, across this study and two previous studies (F. McDougall et al. 2023; F. K. McDougall and Power 2025), 21 tEPEC strains carrying 17 different *bfpA* allele types have now been identified in the five *Pteropus* spp. found on mainland Australia and its external territory of Christmas Island.

The majority of *Pteropus* tEPEC strains were only detected in one bat species, suggesting these tEPEC strains may be *Pteropus‐*species‐specific. However, two strains were shared by different *Pteropus* spp., indicating inter‐species transmission is possible. Such transmission may occur when multiple *Pteropus* spp. are co‐housed in‐care or share roost locations (Timmiss et al. 2021); however, neither scenario occurred with the *Pteropus* spp. sharing tEPEC strains in this study. This finding suggests these two tEPEC strains are not *Pteropus‐*species‐specific and are circulating between different *Pteropus* spp.

The unusual phylogroup G tEPEC strain (ST7791 ONT:H28), first detected in a captive GHFF from NSW in 2015 (F. K. McDougall and Power 2025) and detected here in 16 GHFF individuals from SA in 2018, indicates that the ST7791 ONT:H28 tEPEC strain has been circulating within the GHFF meta‐population for at least 3 years. An additional six *Pteropus* tEPEC strain types were detected in bats at different locations over time periods of up to 2 years, further demonstrating that bat‐specific tEPEC strains are circulating within Australian *Pteropus* populations.

There are several instances where different tEPEC strains share the same *bfpA* allele type, with some of these strains also carried by different *Pteropus* spp., indicating the pEAF plasmid is shared between different bat tEPEC strains and transmission is occurring between different *Pteropus* spp. Notably, one *bfpA* allele (FF‐2.1) was present in three tEPEC strains from GHFF and CIFF, two *Pteropus* species which do not have overlapping ranges and have been geographically isolated for at least 1 million years (Phalen et al. 2017; Tsang et al. 2020). This finding suggests that the FF‐2.1 *bfpA* allele (and associated pEAF plasmid) has been circulating within *Pteropus* spp. for a very long period of time.

There are occasional reports of sporadic infections or low prevalence of tEPEC in non‐human hosts, including domestic animals (cattle, cats, dogs, lambs and pigs) (Jubeda et al. 2014; Blanco et al. 2005; Chandran and Mazumder 2013; Goffaux et al. 2000; Nakazato et al. 2004; Stephan et al. 2004; Wani et al. 2009), wild and captive birds (Borges et al. 2017; Gioia‐Di Chiacchio et al. 2018; Saidenberg et al. 2012; Sanches et al. 2017) and mammals (boar, coyote and deer) (Alonso et al. 2017; Chandran and Mazumder 2013; Ishii et al. 2007). In cases where the tEPEC strain type was determined in non‐human non‐bat animal hosts, the majority of isolates were human‐associated tEPEC strains, indicating the direction of transmission was from human‐hosts to non‐human hosts (Alonso et al. 2017; Borges et al. 2017; V. d. Carvalho et al. 2007; V. M. Carvalho et al. 2003; Gioia‐Di Chiacchio et al. 2018; Nakazato et al. 2004). Across all five Australian *Pteropus* spp. examined to date, the estimated true prevalence of tEPEC was much higher (range 14.2%–37.0%) than tEPEC reports in non‐bat mammal species, with the highest being 5.8% in wild coyotes (Chandran and Mazumder 2013). Additionally, tEPEC were detected in five *Pteropus* spp. over extended time periods ranging from 6 months to 4 years. The high prevalence and consistent presence of tEPEC in five Australian *Pteropus* spp., including one geographically isolated species, further demonstrates that all Australian *Pteropus* spp. are natural tEPEC hosts.

Almost a quarter (24.1%) of *Pteropus bfpA* types were shared by one or more *Pteropus* spp., across large geographical ranges and in multiple years. Within mainland Australia, there is considerable range overlap between the four *Pteropus* spp. (Figure 1) and also considerable roost overlap (Timmiss et al. 2021). A study of *Pteropus* roosts across Australia between 2012 and 2017 found 61.7% of all roosts contained mixed species (Timmiss et al. 2021). The two most frequently observed combinations in mixed‐roosts were GHFF and BFF (124 roosts) and BFF, GHFF and LRFF (94 roosts), and all four species were present in one roost (Timmiss et al. 2021). Mainland *Pteropus* spp. are also extremely mobile, with individuals travelling long distances to forage and frequently moving between roosts (Parsons et al. 2011; Welbergen et al. 2020). The combination of mixed‐species‐roosts and highly mobile individuals establishes transmission routes for tEPEC and pEAF plasmids between the four mainland species of Australian *Pteropus* across their range and within sympatric roosts. Additionally, the frequent co‐housing of multiple *Pteropus* spp. within bat rehabilitation facilities where they aggregate in mixed groups, enhances the potential for direct transfer of tEPEC and pEAF plasmids between individual bats of different species (Mühldorfer 2013).

Whilst range overlaps, mixed‐roosts and co‐housing in‐care may explain the shared distribution of tEPEC strains and *bfpA* types in the four mainland *Pteropus* spp., this explanation does not extend to the geographically isolated CIFF. Phylogenetic analysis determined that CIFF are most closely related to the subclade of *Pteropus hypomelanus* originating from Pulau Panjang Island, located off the northeast coast of Java and approximately 1700 km northwest of Christmas Island (Phalen et al. 2017). Although CIFF and the four mainland *Pteropus* spp., all belong to a clade containing Pacific Ocean islands and mainland Australia *Pteropus* spp., there has been no direct contact between CIFF and mainland species for at least 1 million years (Phalen et al. 2017; Tsang et al. 2020).

The detection of 19 new *bfpA* allele types in this study has expanded the total number of *bfpA* allele types identified in *Pteropus* spp. to 29. The inferred phylogeny of all *Pteropus* and human/non‐bat *bfpA* types revealed that 86% of *Pteropus bfpA* types belonged to distinct bat‐specific lineages (clades FF‐1 to FF‐14). Thirteen of the 14 bat‐specific *bfpA* clades were most closely related to human‐associated beta *bfpA* allele types, but interestingly, the novel FF‐7 *Pteropus bfpA* types belonged to a distinct clade placed between the beta *bfpA* clade and the human‐associated alpha *bfpA* clade. These findings confirm that *Pteropus* spp. have evolved bat‐specific *bfpA* lineages and indicate that *bfpA* has evolved in both humans and *Pteropus* spp. The identification of bat‐specific *bfpA* lineages is also in agreement with a previous study which determined that bat and human *bfp* operon variants belong to separate lineages, but share an evolutionary origin (F. K. McDougall and Power 2025).

## 5 Conclusions

This study has confirmed that all five Australian *Pteropus* spp. are natural tEPEC hosts and that diverse bat‐specific tEPEC strains and novel *bfpA* lineages have evolved in these *Pteropus* spp. Additionally, the consistent detection of tEPEC in five *Pteropus* spp. over a period of 6 years and across vast geographical ranges demonstrates that these bat‐specific tEPEC and *bfpA* lineages have been circulating within and between Australian *Pteropus* spp. for an extremely long time.

Given that tEPEC can no longer be considered a highly adapted pathogen that specifically infects humans, it is also plausible that *Pteropus* spp. represent the evolutionary origin of tEPEC in humans. Previously, bat‐specific tEPEC have demonstrated functional *bfp* operons in HEp‐2 adhesion assays (F. McDougall et al. 2023), thus, further work is warranted to determine if bat‐specific tEPEC strains are zoonotic and capable of infecting humans that share environments with *Pteropus* spp.

## Author Contributions

**Fiona K McDougall:** conceptualisation, methodology, data curation, formal analysis, investigation, funding acquisition, visualisation, project administration, resources, writing – original draft, writing – review and editing. **Laura A Pulscher:** investigation, resources, data curation; writing – review and editing. **Wayne S J Boardman:** investigation, data curation; resources, writing – review and editing. **Karrie Rose:** resources, writing – review and editing, supervision. **Mariel Fulham:** formal analysis, writing – review and editing. **Michelle L Power:** conceptualisation, methodology, investigation, resources, writing – review and editing, funding acquisition, project administration, supervision.

## Ethics Statement

Opportunistic sample collection from bats in wildlife care and on‐going bat programs on mainland Australia was approved by institutional Animal Care and Ethics Committees; Macquarie University (ARA 2017/013) and under a NSW Government Scientific Licence (No. SL101898). Bat capture and sampling in QLD and SA was approved by The University of Adelaide (No. S‐2015‐028) and CSIRO (No. 2016–17), and under SA Department of Environment and Water Wildlife Scientific Permit (M‐23671‐1, 2 and 3) and Queensland Government Scientific Permit (173(P)). Sampling and capture of Christmas Island flying foxes was approved by the Animal Care and Ethics Committee of Western Sydney University (Nos. A11140 & A12791) and under Christmas Island National Park Permits (Nos. CINP_2015‐16_1 and CINP_2018_2) and an Australian Government Environment Protection and Biodiversity Conservation Regulations 2000 license (No AU‐COM2018‐414). Christmas Island flying fox samples were imported under permits granted by the Australian Government Department of Agriculture (Permit Nos. IP15007146 & IP1368078) and transferred to Macquarie University under two Approval to Transfer Biosecurity Material permits granted by the Australian Government Department of Agriculture (Transfer approvals No. 2018/030 and No. T2018/037).

## Conflicts of Interest

The authors declare no conflicts of interest.

## Supporting information


**Supporting Figure S1:** tEPEC in five bat spp_MO_FINAL.

**Supporting Tables S1:** tEPEC in five bat spp_MO_FINAL.

**Supporting Data 1:** Individual tEPEC isolate and faecal DNA sample data.

## Data Availability

The data that support the findings of this study are openly available in GenBank at https://www.ncbi.nlm.nih.gov. DNA sequences for representative flying fox *bfpA* allele variants have been uploaded to GenBank under accession numbers PV285308 to PV285309, PV2853011, PV285314 to PV285327 and PV285329 to PV285349 (individual accession numbers are listed in Table S4).
