# Supplementary material for: Characterisation of Typical Enteropathogenic Escherichia coli (tEPEC) and bfpA Genes From Five Bat Species Reveals Australian Pteropus Spp. Are Natural tEPEC Hosts
Source: Microbiologyopen. 2026 Sep 7;15(5):e70403. doi: 10.1002/mbo3.70403 (PMC13550759; doi:10.1002/mbo3.70403)
Supplement: Supplementary file 1 [file MBO3-15-e70403-s002.pdf]

Supplementary Tables

Title:

Characterisation of typical enteropathogenic *Escherichia coli* (tEPEC) and *bfpA* genes from five bat species reveals Australian *Pteropus* spp. are natural tEPEC hosts

Author names

Fiona K McDougall[1], Laura Pulscher[2,3], Wayne S.J Boardman[4], Karrie Rose[5], Mariel Fulham[1] and Michelle L Power[1]

Author affiliations

[1] School of Natural Sciences, Faculty of Science and Engineering, Macquarie University, Sydney, NSW 2109, Australia

[2] School of Veterinary Science, Faculty of Science, University of Sydney, NSW 2006, Australia

[3] Department of Microbiology, Immunology, and Pathology, Colorado State University, Fort Collins, CO 80521, United States

[4] School of Animal and Veterinary Sciences, College of Sciences, Adelaide University, Roseworthy, SA 5371, Australia

[5] Australian Registry of Wildlife Health, Taronga Conservation Society Australia, Bradleys Head Road, Mosman, NSW 2088, Australia

Table S1. Typical Enteropathogenic *Escherichia coli* (tEPEC) cultured from flying fox faecal samples. BFF; Black flying fox. CIFF; Christmas Island flying fox. GHFF; Grey headed flying fox. LRFF; Little red flying fox. SFF; Spectacled flying fox. NSW; New South Wales. QLD; Queensland. SA; South Australia.

**Sample collection date**	***Pteropus* species**	**Location of sampling**	**Wild / In-care**	**Adults / Pups**	**No. samples cultured for *E. coli***	**tEPEC culture study reference**	**No. tEPEC characterised in this study**	**No. full length *bfpA* sequences obtained**
Apr 2015	BFF	Tolga Bat Hospital, QLD	In-care	Unknown	1	This study	0	0
Apr 2018	BFF	Coen, QLD	Wild-caught	Unknown	9	This study	0	0
Apr 2018	BFF	Burketown, QLD	Wild-caught	Unknown	5	This study	0	0
Aug 2018	BFF	Charters Towers, QLD	Wild-caught	Unknown	5	This study	0	0
Dec 2018 to Jan 2019	BFF	Northern NSW	In-care	Pups	7	This study	1	1
Jan 2019	BFF	Brisbane/Sunshine Coast, QLD	In-care	Pups	13	This study	0	0
Mar 2019	BFF	Sydney, NSW	In-care	Pups	1	This study	0	0
Oct 2019	BFF	Coen, QLD	Wild-caught	Unknown	4	This study	0	0
	Total BFF				45		1	1
Jun 2018 to Mar 2019	CIFF	Christmas Island	Wild-caught	Adults and pups	58	This study	6	6
Feb 2015 to Jul 2015	GHFF	Matcham, NSW	In-care	Adults	44	This study	0	0
Apr 2015	GHFF	Tolga Bat Hospital, QLD	In-care	Adult	1	This study	0	0
Feb 2017	GHFF	Adelaide, SA	Wild-caught	Adults	46	(1)	13	13
Feb 2018	GHFF	Adelaide, SA	Wild-caught	Adults	52	(1)	13	13
Feb 2018	GHFF	Mylor, SA	In-care	Adults	20	This study	15	15
Apr 2018	GHFF	Blackalls Park, NSW	Wild-opportunistic	Adults	51	(1)	2	2
Oct 2018 to Apr 2019	GHFF	Sydney and Shoalhaven, NSW	In-care	Pups	25	This study	1	1
Nov 2018 to Jan 2019	GHFF	Adelaide and Mylor, SA	In-care	Pups	53	This study	0	0
Jan 2019	GHFF	Albany Creek, QLD	In-care	Pups	1	This study	0	0
Apr 2019	GHFF	Blackalls Park, NSW	Wild-opportunistic	Adults	71	(1)	5	5
Mar 2021	GHFF	Adelaide, SA	In-care	Pups	30	This study	0	0
	Total GHFF				394		49	49
Apr 2015	LRFF	Tolga Bat Hospital, QLD	In-care	Unknown	7	This study	0	0
Aug 2018	LRFF	Charters Towers, QLD	Wild-caught	Unknown	5	This study	0	0
Sep 2018	LRFF	Charters Towers, QLD	Wild-caught	Unknown	7	This study	0	0
Dec 2018	LRFF	Mount Isa, QLD	Wild-caught	Unknown	10	This study	1	1
Jan 2019	LRFF	Woodford, QLD	Wild-caught	Unknown	5	This study	0	0
Oct 2019	LRFF	Coen, QLD	Wild-caught	Unknown	11	This study	1	1
	Total LRFF				53		2	2
Apr 2015	SFF	Tolga Bat Hospital, QLD	In-care	Unknown	14	This study	0	0
Nov 2018	SFF	Cairns, QLD	Wild-opportunistic	Adults	10	This study	0	0
Dec 2018	SFF	Tolga Bat Hospital, QLD	In-care	Pups	16	This study	0	0
Mar 2019	SFF	Gordonvale, QLD	Wild-caught	Unknown	8	This study	5	5
	Total SFF				40		5	5
**Totals - All flying foxes**					**590**		**63**	**63**

Table S2. Faecal DNA samples collected from five species of flying foxes (*Pteropus* bats) and screened for typical Enteropathogenic *E. coli* (tEPEC) using PCR (*eae/bfpA/chuA)* in this study. BFF; Black flying fox. CIFF; Christmas Island flying fox. GHFF; Grey headed flying fox. LRFF; Little red flying fox. SFF; Spectacled flying fox. NSW; New South Wales. QLD; Queensland. SA; South Australia.

Sample collection date	*Pteropus* species	Location of sampling	Wild /In-care	Adults / Pups	No. faecal samples DNA extracted	DNA extraction study reference	No. tEPEC positive (*eae/bfpA* PCR)	No. full length *bfpA* allele types obtained from faecal DNA
Apr 2015	BFF	Tolga Bat Hospital, QLD	In-care	Unknown	1	(2)	0	0
2016 to 2017	BFF	Biosecurity Laboratory, QLD	Wild-necropsy	Unknown	14	This study	2	2
Apr 2018	BFF	Coen, QLD	Wild-caught	Unknown	9	This study	2	2
Apr 2018	BFF	Burketown, QLD	Wild-caught	Unknown	5	This study	1	1
Aug 2018	BFF	Charters Towers, QLD	Wild-caught	Unknown	5	This study	0	0
Dec 2018 to Jan 2019	BFF	Northern NSW	In-care	Pups	7	This study	3	2
Jan 2019	BFF	Brisbane/Sunshine Coast, QLD	In-care	Pups	13	This study	0	0
Mar 2019	BFF	Sydney, NSW	In-care	Pups	1	This study	0	0
Oct 2019	BFF	Coen, QLD	Wild-caught	Unknown	4	This study	0	0
	Total BFF				59		8	7
Jun 2018 to Mar 2019	CIFF	Christmas Island	Wild-caught	Adults and pups	24	This study	8	3
Feb 2015 to Jul 2015	GHFF	Wambina, Matcham, NSW	In-care	Adults	69	(2)	5	4
Apr 2015	GHFF	Tolga Bat Hospital, QLD	In-care	Adult	1	(2)	0	0
Feb 2018	GHFF	Mylor, SA	In-care	Adults	20	This study	17	1
Oct 2018 to Apr 2019	GHFF	Sydney and Shoalhaven, NSW	In-care	Pups	24	(3)	5	4
Nov 2018 to Jan 2019	GHFF	Adelaide and Mylor, SA	In-care	Pups	53	(3)	10	7
Jan 2019	GHFF	Albany Creek, QLD	In-care	Pups	1	(3)	0	0
Jan 2020	GHFF	Sydney and Central Coast, NSW	In-care	Pups	11	(3)	8	7
Mar 2021	GHFF	Adelaide, SA	In-care	Pups	30	(3)	6	4
	Total GHFF				208		51	28
Apr 2015	LRFF	Tolga Bat Hospital, QLD	In-care	Unknown	8	(2)	6	4
2016 to 2017	LRFF	Biosecurity Laboratory, QLD	Wild-opportunistic	Unknown	2	This study	0	0
Aug 2018	LRFF	Charters Towers, QLD	Wild-caught	Unknown	5	This study	2	2
Sep 2018	LRFF	Charters Towers, QLD	Wild-caught	Unknown	7	This study	1	1
Dec 2018	LRFF	Mount Isa, QLD	Wild-caught	Unknown	10	This study	4	3
Jan 2019	LRFF	Woodford, QLD	Wild-caught	Unknown	5	This study	3	0
Oct 2019	LRFF	Coen, QLD	Wild-caught	Unknown	11	This study	1	1
	Total LRFF				48		17	11
Apr 2015	SFF	Tolga Bat Hospital, QLD	In-care	Unknown	12	(2)	5	2
2016 to 2017	SFF	Biosecurity Laboratory, QLD	Wild-opportunistic	Unknown	1	This study	0	0
Mar 2019	LRFF	Gordonvale, QLD	Wild-caught	Unknown	8	This study	6	1
Nov 2018	SFF	Cairns, QLD	Wild-opportunistic	Adults	10	This study	4	3
Dec 2018	SFF	Tolga Bat Hospital, QLD	In-care	Pups	16	This study	1	1
	Total SFF				47		16	7
**Totals - All flying foxes**					**386**		**100**	**56**

Table S3. Reference sequences for *bfpA* allele typing (n = 28).

***bfpA* alleles**	**GenBank accession No.**	**tEPEC host**	**Reference**
α1 (alpha-1)	AF304474, AF304480	Human	(4)
α2 (alpha-2)	AF304469, AF304473, AF304477, AF304485	Human	(4)
a3 (alpha-3)	AF304468, AF304470, AF304472, AF304484	Human	(4)
a5 (alpha-5)	FN391182	Human	Blanco et al., Unpublished
ß1 (beta-1)	AF304471	Human	(4)
ß1.2 (beta-1)	OM925979	*Pteropus* bat	(1)
ß2 (beta-2)	AF304486	Avian	(4)
ß3 (beta-3)	AF304476	Canine	(4)
ß4 (beta-4)	AF304478	Human	(4)
ß5 (beta-5)	AF304475, AF304479, AF304482	Human	(4)
ß6 (beta-6)	AF474407	Human	(5)
ß7.1 (beta-7.1)	EF011024	Human	(6)
ß7.2 (beta-7.2)	EF011025, EF011026	Human	(6)
ß8 (beta-8)	FN391180	Human	Blanco et al., Unpublished
ß10 (beta-10)	FN391178	Bovine	Blanco et al., Unpublished
ß11 (beta-11)	FN391179	Human	Blanco et al., Unpublished
ß12 (beta-12)	AB247934	Human	(7)
ß13 (beta-13)	AB247926	Human	(7)
ß14 (beta-14)	AB247933	Human	(7)
ß14.2 (beta-14.2)	OM925980, PV285310	*Pteropus* bat	(1)
FF-1.1	OM925981, OM925982	*Pteropus* bat	(1)
FF-1.2	PV285312	*Pteropus* bat	(8)
FF-2.1	OM925983	*Pteropus* bat	(1)
FF-3.1	OM925984	*Pteropus* bat	(1)
FF-4.1	OM925985	*Pteropus* bat	(1)
FF-4.2	OM925986, PV285328	*Pteropus* bat	(1)
FF-5.1	OM925987	*Pteropus* bat	(1)
FF-6.1	PV285313	*Pteropus* bat	(8)

Table S4. GenBank accessions numbers for full length flying fox 27 *bfpA* allele types identified in five Australian flying fox (*Pteropus* bat) species and BLASTn searches of closest matches to GenBank *bpfA* alleles. tEPEC, Typical enteropathogenic *Escherichia coli*.

**Bat *bfpA* allele type**	**GenBank Accession number(s)**	**Source - tEPEC isolate and/or faecal DNA (Number of detections)**	**GenBank Accession No. of closest match**	**Closest *bfpA* reference or undefined allele**	**Identity %**
beta-1.2	PV285308	tEPEC isolates (4)	AF304471	beta-1	99.15%
beta-7.3	PV285309	Faecal DNA (1)	EF011025	beta-7.2	99.83%
beta-14.3	PV285311	Faecal DNA (1)	AB247933	beta-14	98.83%
FF-1.1	PV285314, PV285315	tEPEC isolates (6), Faecal DNA (1)	FN391180	beta-8	92.69%
FF-1.2	PV285316	Faecal DNA (1)	FN391180	beta-8	92.86%
FF-1.3	PV285317	tEPEC isolates (2)	FN391180	beta-8	92.52%
FF-1.4	PV285318	Faecal DNA (1)	FN391180	beta-8	92.70%
FF-2.1	PV285319 – PV285322	tEPEC isolates (7), Faecal DNA (10)	CP117640	Not defined	88.33%
FF-3.1	PV285322 – PV285325	tEPEC isolate (1), Faecal DNA (3)	FN391180	beta-8	90.51%
FF-4.1	PV285326, PV285327	tEPEC isolates (7), Faecal DNA (2)	FN391180	beta-8	93.54%
FF-4.3	PV285329	tEPEC isolate (1)	CP117640	Not defined	93.54%
FF-4.4	PV285330	Faecal DNA (1)	CP117640	Not defined	93.71%
FF-5.1	PV285331 – PV285333	tEPEC isolates (3), Faecal DNA (9)	AF304476	beta-3	93.71%
FF-6.1	PV285334	tEPEC isolates (16), Faecal DNA (3)	FN391180	beta-8	90.19%
FF-6.2	PV285335	tEPEC isolate (1)	FN391180	beta-8	90.07%
FF-7.1	PV285336	tEPEC isolates (2)	AF304485<br>AF304484	alpha-2<br>alpha-3	96.91%<br>96.91%
FF-7.2	PV285337	Faecal DNA (1)	AF304484	alpha-3	96.91%
FF-7.3	PV285338	Faecal DNA (3)	AF304485<br>AF304484	alpha-2<br>alpha-3	97.08%<br>97.08%
FF-7.4	PV285339	Faecal DNA (6)	AF304485<br>AF304484	alpha-2<br>alpha-3	96.91%<br>96.91%
FF-8.1	PV285340	tEPEC isolate (1)	FN391180	beta-8	91.16%
FF-9.1	PV285341	tEPEC isolates (9), Faecal DNA (1)	FN391180	beta-8	93.54%
FF-9.2	PV285342	Faecal DNA (1)	FN391180	beta-8	93.26%
FF-10.1	PV285343, PV285344	tEPEC isolates (3), Faecal DNA (2)	AF304484	alpha-3	92.36%
FF-11.1	PV285345	Faecal DNA (1)	AB247933	beta-14	93.47%
FF-12.1	PV285346	Faecal DNA (1)	EF011025	beta-7.2	90.80%
FF-13.1	PV285347, PV285348	Faecal DNA (6)	CP117640	Not defined	95.68%
FF-14.1	PV285349	Faecal DNA (1)	AF304485<br>AF304484	alpha-2<br>alpha-3	91.30%<br>91.30%

Table S5. Individual tEPEC isolate NCBI SRA BioProject and Biosample IDs, and EnteroBase Barcodes for *Pteropus* tEPEC reference strains. GHFF; Grey headed flying fox. SFF; Spectacled flying fox. WGS; Whole genome sequencing.

Isolate ID	Host species	Wild/ In-care	ST	Serotype	*bfpA* allele type	Phylogroup (WGS)	tEPEC clade	Bioproject_ID	Biosample ID	Enterobase Barcode	Reference
FF593	GHFF	In-care	7791	ONT:H28	FF-6.1	G	F	PRJNA613581	SAMN47229884	ESC_HA7318AA	(8)
FF618	SFF	In-care	1041	ONT:H39	FF-1.2	B2	E	PRJNA613581	SAMN47229885	ESC_HA7322AA	(8)
FF773	GHFF	Wild	7797	ONT:H1	FF-2.1	B2	A	PRJNA613581	SAMN14409778	ESC_HA7323AA	(1)
FF781	GHFF	Wild	7799	ONT:H1	FF-4.1	B2	A	PRJNA613581	SAMN14409779	ESC_HA7324AA	(1)
FF782	GHFF	Wild	583	O71:H6	FF-3.1	B2	C	PRJNA613581	SAMN14409780	ESC_RA4667AA	(1)
FF787	GHFF	Wild	7799	ONT:H51	FF-1.1	B2	A	PRJNA613581	SAMN14409781	ESC_HA7325AA	(1)
FF796	GHFF	Wild	7799	ONT:H51	FF-1.1	B2	A	PRJNA613581	SAMN14409784	ESC_HA7328AA	(1)
FF802	GHFF	Wild	7796	O118/O151:H34	beta-1.2	B2	C	PRJNA613581	SAMN14409785	ESC_HA7329AA	(1)
FF812	GHFF	Wild	7799	ONT:H51	FF-1.1	B2	A	PRJNA613581	SAMN14409786	ESC_HA7330AA	(1)
FF831	GHFF	Wild	2346	ONT:H45	FF-5.1	B2	A	PRJNA613581	SAMN14409788	ESC_RA4669AA	(1)
FF841	GHFF	Wild	2346	O109:H45	FF-5.1	B2	A	PRJNA613581	SAMN14409789	ESC_RA4670AA	(1)
FF851	GHFF	Wild	2346	ONT:H45	FF-5.1	B2	A	PRJNA613581	SAMN14409790	ESC_RA4668AA	(1)
FF1136	GHFF	Wild	9732	O48:H1	FF-4.2	B2	A	PRJNA613581	SAMN14409807	ESC_JA9913AA	(1)
FF1146A	GHFF	Wild	8437	O180:H34	FF-1.1	B2	A	PRJNA613581	SAMN14409808	ESC_JA9911AA	(1)
FF1172	GHFF	Wild	9731	O91:H40	beta-14.2	B2	D	PRJNA613581	SAMN14409800	ESC_JA9917AA	(1)

References for supplementary tables

1. McDougall F, Gordon D, Robins-Browne R, Bennett-Wood V, Boardman WS, Graham PL, Power M. Characterisation of typical enteropathogenic *Escherichia coli* (tEPEC) lineages and novel *bfpA* variants detected in Australian fruit bats (*Pteropus poliocephalus*). *Sci Total Environ*. 2023;902:166336.

2. Schiller, S. E., Webster, K. N., & Power, M. (2016). Detection of *Cryptosporidium hominis* and novel *Cryptosporidium* bat genotypes in wild and captive *Pteropus* hosts in Australia. *Infection, Genetics and Evolution*, *44*, 254-260.

3. McDougall, F. K., Speight, N., Funnell, O., Boardman, W. S., & Power, M. L. (2024). Dynamics of Antimicrobial Resistance Carriage in Koalas (*Phascolarctos Cinereus*) and Pteropid Bats (*Pteropus Poliocephalus)* Before, During and After Wildfires. *Microbial Ecology*, *87*(1), 39.

4. Blank TE, Zhong H, Bell AL, Whittam TS, Donnenberg MS. Molecular variation among type IV pilin (*bfpA*) genes from diverse enteropathogenic *Escherichia coli* strains. *Infect Immun*. 2000;68(12):7028-38.

5. Blank TE, Lacher DW, Scaletsky IC, Zhong H, Whittam TS, Donnenberg MS. Enteropathogenic *Escherichia coli* O157 strains from Brazil. *Emerg Infect Dis*. 2003;9(1):113.

6. Lacher DW, Steinsland H, Blank TE, Donnenberg MS, Whittam TS. Molecular evolution of typical enteropathogenic *Escherichia coli*: clonal analysis by multilocus sequence typing and virulence gene allelic profiling. *J Bacteriol*. 2007;189(2):342-50.

7. Iida M, Yamazaki M, Yatsuyanagi J, Ratchtrachenchai OA, Subpasu S, Okamura N, Ito K. Typing of *bfpA* genes of enteropathogenic *Escherichia coli* isolated in Thailand and Japan by heteroduplex mobility assay. *Microbiol immunol*. 2006;50(9):713-7.

8. McDougall, F. K., & Power, M. L. (2025). Novel strains of typical enteropathogenic *Escherichia coli* (tEPEC) in *Pteropus* bats, including the first report of a phylogroup G tEPEC. *Microbial Genomics*, *11*(8), 001469.
