# Supplementary material for: Characterisation of Typical Enteropathogenic Escherichia coli (tEPEC) and bfpA Genes From Five Bat Species Reveals Australian Pteropus Spp. Are Natural tEPEC Hosts
Source: Microbiologyopen. 2026 Sep 7;15(5):e70403. doi: 10.1002/mbo3.70403 (PMC13550759; doi:10.1002/mbo3.70403)
Supplement: Supplementary file 2 [file MBO3-15-e70403-s003.pdf]

Supplementary Figure 1

Title:

Characterisation of typical enteropathogenic *Escherichia coli* (tEPEC) and *bfpA* genes from five bat species reveals Australian *Pteropus* spp. are natural tEPEC hosts

Author names

Fiona K McDougall[1], Laura A Pulscher[2,3], Wayne S.J Boardman[4], Karrie Rose[5], Mariel Fulham[1] and Michelle L Power[1]

Author affiliations

[1] School of Natural Sciences, Faculty of Science and Engineering, Macquarie University, Sydney, NSW 2109, Australia

[2] School of Veterinary Science, Faculty of Science, University of Sydney, NSW 2006, Australia

[3] Department of Microbiology, Immunology, and Pathology, Colorado State University, Fort Collins, CO 80521, United States

[4] School of Animal and Veterinary Sciences, College of Sciences, Adelaide University, Roseworthy, SA 5371, Australia

[5] Australian Registry of Wildlife Health, Taronga Conservation Society Australia, Bradleys Head Road, Mosman, NSW 2088, Australia

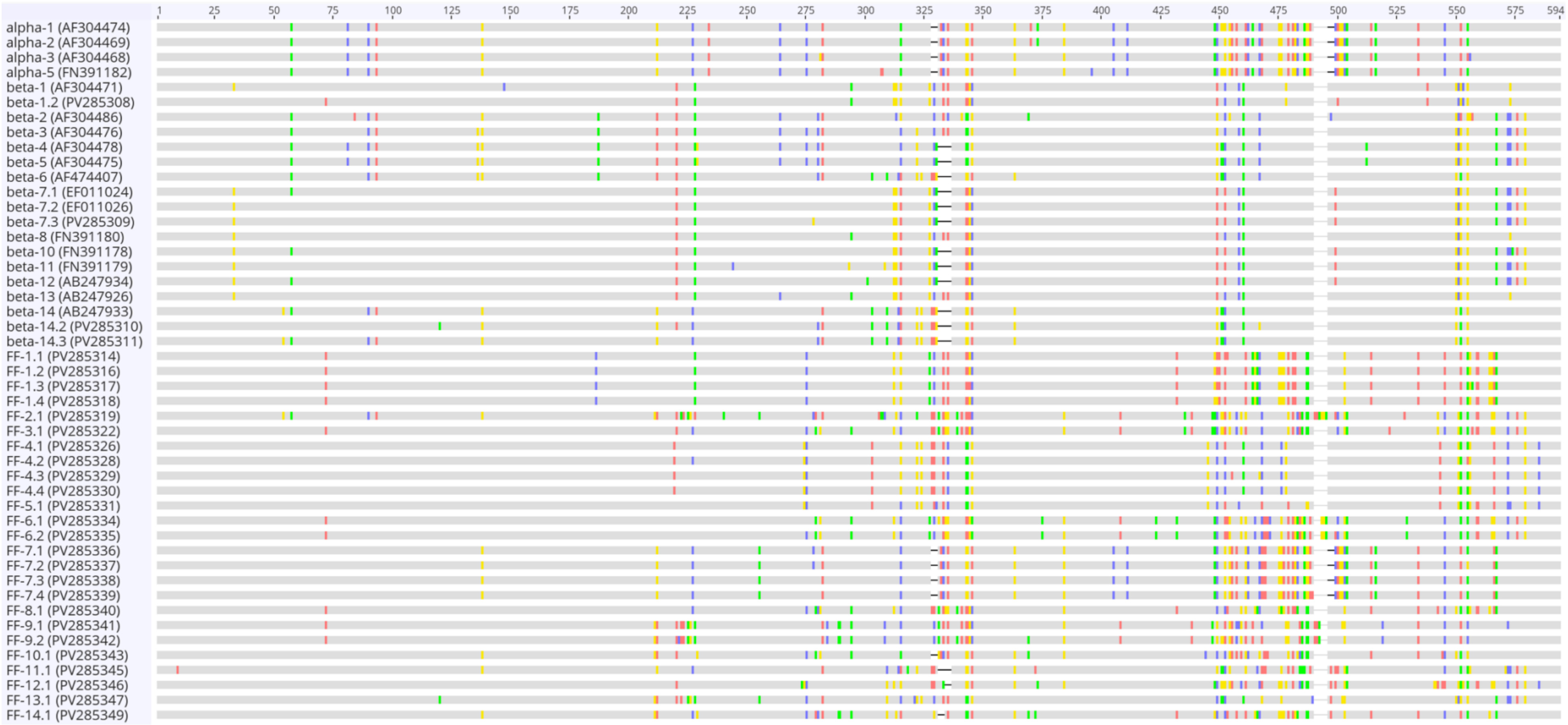

Figure S1. Nucleotide alignment of all *Pteropus bfpA* allele sequences (*n* = 27; Table S4) and 20 reference *bfpA* alleles (human *n* = 15, non-bat animal *n* = 3 and flying foxes in previous studies *n* = 2, beta-14.2 and FF-4.2) (Table S3). Sequence labels indicate *bfpA* variant and corresponding GenBank accession number.
